# Spatial flocking: Control by speed, distance, noise and delay

**DOI:** 10.1371/journal.pone.0191745

**Published:** 2018-05-04

**Authors:** Illés J. Farkas, Shuohong Wang

**Affiliations:** 1 Department of Automation Control, Huazhong University of Science and Technology, 1037 Luoyu Rd, Wuhan, China, 430074; 2 MTA-ELTE Statistical and Biological Physics Research Group, Hungarian Academy of Sciences, Pázmány Péter sétány 1A, Budapest, Hungary, 1117; 3 School of Computer Science, Fudan University, 825 Zhangheng Rd, Shanghai, China, 201203; Rijksuniversiteit Groningen, NETHERLANDS

## Abstract

Fish, birds, insects and robots frequently swim or fly in groups. During their three dimensional collective motion, these agents do not stop, they avoid collisions by strong short-range repulsion, and achieve group cohesion by weak long-range attraction. In a minimal model that is isotropic, and continuous in both space and time, we demonstrate that (i) adjusting speed to a preferred value, combined with (ii) radial repulsion and an (iii) effective long-range attraction are sufficient for the stable ordering of autonomously moving agents in space. Our results imply that beyond these three rules ordering in space requires no further rules, for example, explicit velocity alignment, anisotropy of the interactions or the frequent reversal of the direction of motion, friction, elastic interactions, sticky surfaces, a viscous medium, or vertical separation that prefers interactions within horizontal layers. Noise and delays are inherent to the communication and decisions of all moving agents. Thus, next we investigate their effects on ordering in the model. First, we find that the amount of noise necessary for preventing the ordering of agents is not sufficient for destroying order. In other words, for realistic noise amplitudes the transition between order and disorder is rapid. Second, we demonstrate that ordering is more sensitive to displacements caused by delayed interactions than to uncorrelated noise (random errors). Third, we find that with changing interaction delays the ordered state disappears at roughly the same rate, whereas it emerges with different rates. In summary, we find that the model discussed here is simple enough to allow a fair understanding of the modeled phenomena, yet sufficiently detailed for the description and management of large flocks with noisy and delayed interactions. Our code is available at http://github.com/fij/floc.

## Introduction: Collective motion in 2 and 3 dimensions

In all fields of life recent technological developments have lead to a surge in data acquisition. However, usually the obtained data can be put to practical use only with improved analytic and predictive methods. For collective motion (swarming, active matter), some of the recent major experimental advances have been the systematic measurements of fish trajectories in small shoals [1], tracking the individual coordinates of up to 2700 birds in flocks [2], and obtaining GPS track logs of homing pigeons flying together [3]. Initially, experimental and modeling efforts were focused on planar (2 dimensional) motion. Due to these efforts it is now well known that in 2 dimensions bacteria, insects, horses, and also humans display collective motion patterns [4–7]. Compared to planar motion, an agent moving in space can be kept aligned by a higher number of nearest neighbor interactors. At the same time, it has also more directions to turn away from the consensus of those nearest neighbors.

The most straightforward local rule that can describe the alignment of moving agents with their neighbors is to set each agent’s direction of motion explicitly to the average direction of its neighbors [8, 9]. Turning continuously toward the average direction of the neighbors is also possible [10, 11]. More detailed mechanisms of the alignment include anisotropic interactions caused by elongated shapes [12–15], also combined with a frequent reversal of the direction of motion [16], the preference for movements in the horizontal plane (as opposed to vertical movements) [17], a viscous medium [18], friction among the agents and inelastic collisions [19, 20], and sticking together [21]. While these rules can set the direction of motion for the agents, collision avoidance and cohesion (staying together) are also necessary for flock formation. To avoid the collisions of moving and interacting agents the simplest solution is to let all agents (in the model) have zero size [8]. A more realistic solution is a strong short-range repulsive interaction, in which the magnitude of the repulsion force becomes very high when two agents come too close. Finally, for keeping the group together two commonly applied modeling tools are the spatial confinement of the group (e.g., periodic boundaries) and a weak attraction that is turned on when distances between the agents grow.

**Fig 1:**
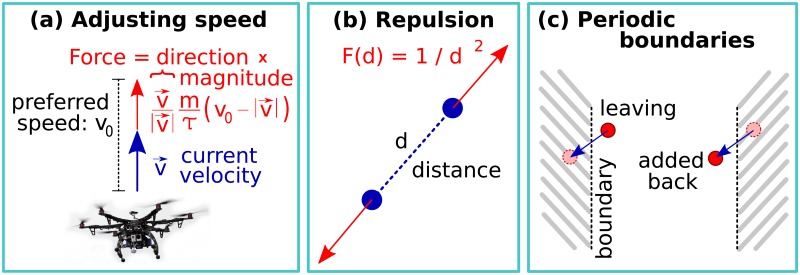
The spatial flocking model used in the paper. Image from thedroneinfo.com.

The model that we discuss here focuses on *controlling the speed of the agents individually*. The speed of an agent is adjusted to the preferred speed with a rate that is proportional to the difference from the preferred value (see Fig 1). This modeling approach is realistic, because—according to recent experimental and modeling evidence—individual *speed control* plays a key role in the formation and the stability of bird flocks and fish shoals [1, 22, 23]. Also, experiments and models for vibrated self-propelled hard disks have shown that the binary collisions caused by *maintaining speed* can align velocity vectors first locally, and then also across the entire system [24].

We investigate the effect of time delay and noise, too. Time delay is a common phenomenon caused by latent communication between agents, information processing cost, and inertial reasons [25, 26]. Noise at all levels is also inherent to the communication and decisions of all moving agents in a dynamic system and can lead to transitions between behavioral patterns [26–29]. Regarding the combination of time delay and noise, simulation results in [30] showed that a system with noise and delay displays bistability of several coherent patterns. Here we investigate both aspects and show their fundamental dissimilarities.

## Model: A minimal continuous description of spatial flocking

Here we investigate the 3-dimensional version of the generic model that was suggested in [31] and analyzed for 2 dimensions in [32]. The model is continuous in both space and time, and it contains *N* agents. The *i*th agent’s position is ${\vec{r}}_{i}\left(t\right)=\left(x_{i}\left(t\right),y_{i}\left(t\right),z_{i}\left(t\right)\right)$ and its velocity is ${\vec{v}}_{i}\left(t\right)=\left(v_{x,i}\left(t\right),v_{y,i}\left(t\right),v_{z,i}\left(t\right)\right)$. During its motion each agent continuously adjusts its speed, *v*_*i*_(*t*), toward a constant preferred *v*_0_ value with characteristic time *τ*. To model collision avoidance, we apply pairwise radial repulsion among the two agents with a magnitude of *F*(*r*) = *cr*^−2^ as a function of their distance, *r* (for correct dimensions we set *c* = 1*Nm*^2^). For simplicity, we set the mass of each agent to *m* = 1*kg* and their time constants for adjusting speed to *τ* = 1*s*. Based on the above, the equations of motion are (for *i* = 1…*N*):

$$m\frac{d{\vec{v}}_{i}}{dt}=\frac{m}{\tau }\frac{{\vec{v}}_{i}}{\left|{\vec{v}}_{i}\right|}\left(v_{0}-\left|{\vec{v}}_{i}\right|\right)-\sum_{j\neq i}\vec{F}\left(\left|{\vec{r}}_{i}-{\vec{r}}_{j}\right|\right).$$ (1)

Note that the first term on the right hand side of Eq 1 points toward the *i*th agent’s own direction of motion, ${\vec{v}}_{i}/\left|{\vec{v}}_{i}\right|$. In other words, this simplified model separates collision avoidance (second term) from keeping the preferred speed (first term).

We solve the equations of motion numerically by applying the midpoint method with an integration time step of Δ*t* = 10^−3^*s* in a cube that has side length *L* = 50*m* and periodic boundary conditions in all three directions. For two particles the precision of the forward Euler method is sufficient. To integrate the equations of motion of many particles we apply the midpoint method. When the simulation is started we place all agents at random positions—but no pair of them is allowed to be closer than 0.6*LN*^−1/3^—and set all speeds to the preferred speed, *v*_0_ = 5*m*/*s*. We start the system either from a disordered state or an ordered state. When starting the system from the disordered state, we set the directions of the velocity vectors to *N* *different*, randomly selected directions at simulation time *t* = 0. When starting from the ordered state, we set all directions to the *same*, randomly selected direction at time *t*_0_ = −10^5^, then simulate the system until *t* = 0, and after that start to log positions and velocities.

The two additional aspects of the model that we investigate are its responses to noise and time delays. We include noise into the model by adding a random $\vec{\xi }$ vector to the right hand side of Eq 1. This vector is uncorrelated both in time and among agents, its direction is distributed uniformly in space, and its magnitude is a constant, *ξ*. If *ξ* = 1, then the autocorrelation of $\vec{\xi }$ is 1, therefore, the noise vector added to the r.h.s. of Eq 1 during a simulation update of length Δ*t* is $\xi \sqrt{\Delta t}$. Similary, for the first half-step of the midpoint update the amplitude of the applied noise vector is $\xi \sqrt{\Delta t/2}$. In addition to the above, we compute interactions with a distance-based upper cutoff: in the simulations two agents interact only if their distance is below a fixed cutoff radius, *R* = 10*m*. Finally, for efficient computation, we apply a 3-dimensional grid with side length *R* and search for an agent’s interactors—i.e., other agents closer than *R*—only within its own grid cell and the neighboring 26 grid cells.

## Results: Alignment and flocking

**Fig 2:**
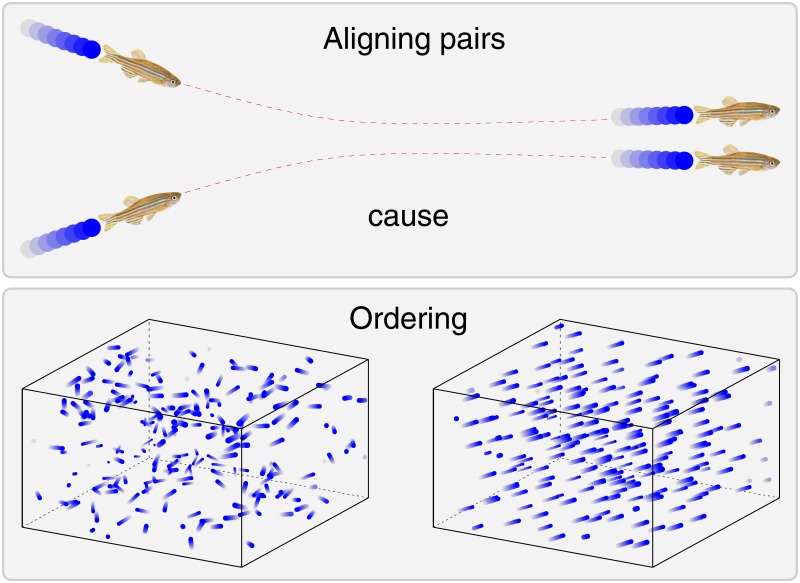
The alignment of pairwise interacting agents can cause large-scale ordering. **Top panel**. In many cases the motion of two interacting agents (e.g., fish, birds or humans) becomes more parallel after their encounter. Here two fish are shown as an illustration, and their paths are indicated by sequences of colored filled disks. Image source: Wikipedia. **Bottom panel**. Paths of *N* = 200 agents for Δ*t* = 0.4 time in an actual simulation—midpoint integration of Eq 1—that was started from a disordered initial state (bottom left) and then reached the ordered state (bottom right).

A necessary condition for the emergence of a single stable aligned group containing the majority of all agents is the ability of small groups to align. More specifically, the most frequently studied necessary condition is whether the alignment of *two* agents increases during their encounter, i.e., when they come close and depart as in the top panel of Fig 2.

### Alignment of two agents in a symmetric encounter

**Fig 3:**
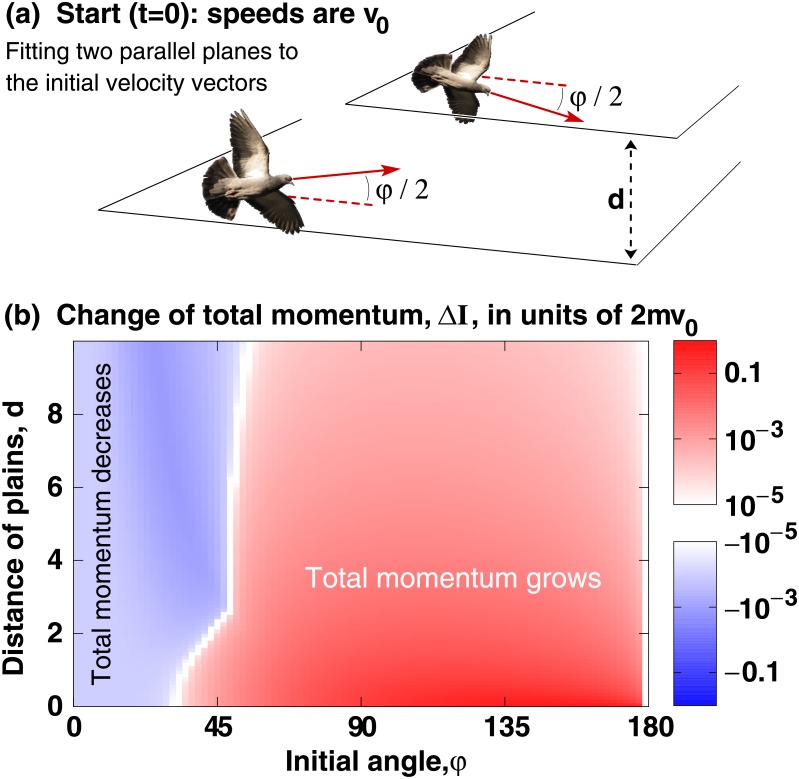
**(a)** Initial state (*t* = 0) of the two symmetrically moving identical agents. Image from openclipart.org. **(b)** Change of the total momentum of the two agents as a function of the initial angle, *φ*, and the distance, *d*, of the planes of their initial velocities. For the major part of the (*d*, *φ*) parameter range the two agents’ total momentum grows during their encounter, i.e., they become more aligned. See text for details.

As a simple—yet nontrivial—case of a two-agent encounter in space, we analyse the scenario when two *identical* agents move such that their velocity vectors are permanently mirror images of each other with respect to the two agents’ center of mass (see Fig 3a). To parametrize this 3-dimensional motion of the two agents, we fit two parallel planes to the skew lines defined by their velocity vectors at *t* = 0. The distance of these two plains is *d*, and both agents have a speed of *v*_0_. When looking at the two agents from the direction perpendicular to the two planes, at *t* = 0 the distance of the two agents is 1,000*m* and the angle of their velocity vectors is *φ*. As in Eq 1, radial repulsion is *F*(*r*) = *cr*^−2^ (with *c* = 1*Nm*^2^ for correct dimensions). We compute this pair encounter by integrating Eq 1 with the forward Euler method with an integration time step of *dt* = 10^−5^, and the parameters *v*_0_ = 1*m*/*s*, *τ* = 1*s* and *ξ* = 0. We stop the integration when the horizontal distance of the agents reaches 1,100*m*. Finally, we obtain an estimate of the error of the numerical integration: we test how the integration time step parameter (*dt*) influences the calculated change of this two-particle system’s total momentum (Δ*I*) during the encounter. We find that the maximal difference of the final Δ*I* values between the *dt* = 10^−4^ and *dt* = 10^−5^ cases is 1.8 × 10^−4^. This difference is significantly smaller than the Δ*I* values in Fig 3 that are all above 10^−3^.

According to Fig 3b, we obtain the following result for the investigated symmetric collision. If the initial angle, *φ*, exceeds a threshold value—which is between 40 and 50 degrees depending on the distance, *d*, of the two particles’ initial planes,—then the total momentum of the two particles increases. In other words, Eq 1 implies that two particles arriving symmetrically at a large angle will depart at a smaller angle: they will become more parallel. With many interacting and moving particles the interactions are usually not pairwise and typically not symmetrical. Therefore, the results displayed in Fig 3b are not more than a significant microscopic result pointing at the possibility of the large-scale alignment of all moving agents. This possibility is tested in Sec below.

### Alignment of many collectively moving agents

**Fig 4:**
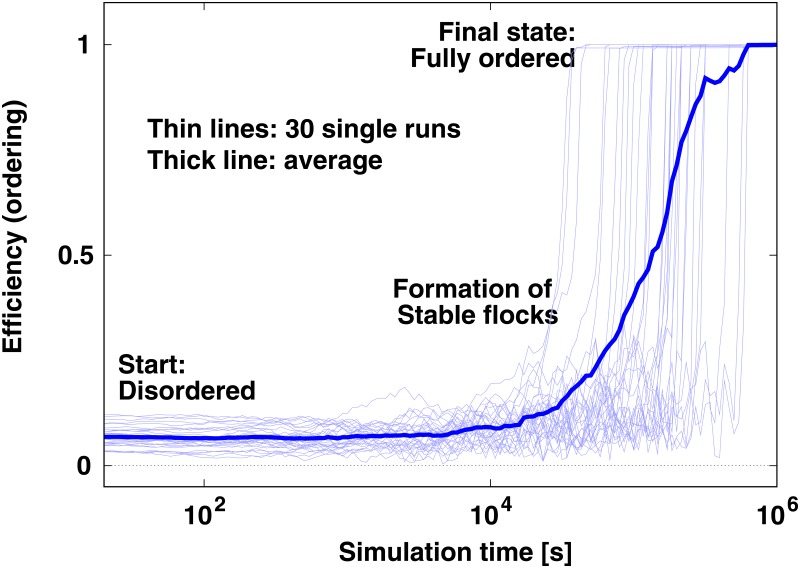
Transition from the initial disordered state (low *E*) to the ordered state (high *E*) in the model of Eq 1 without noise (*ξ* = 0). Note that even without explicit noise (see Eq 1) the noise amplitudes are $\xi \left(t\right)=|{\vec{\xi }}_{i}\left(t\right)|=0$) there are two sources of noise in the simulations: the errors of the numerical integration method and rounding. However, in the present case the numerical integration method is the dominant source of noise.

To test whether Eq 1 can indeed lead to a stable coherent motion of all agents, we start the system from a fully disordered setup and check if it reaches a stable ordered state (see Fig 4). We simulate the motion of agents—i.e., perform the midpoint integration of Eq 1—within a cube that has side length *L* = 50 and periodic boundary conditions. Parameters are *v*_0_ = 5 (preferred speed), *τ* = 1 (time constant for reaching the preferred speed), *R* = 10 (interaction cutoff radius), *dt* = 10^−3^ (time step of integration) and *ξ* = 0 (no noise). At simulation time *t* = 0 the speed of each agent is *v*_0_ and velocity vectors point to independently selected random directions. Starting the system with this scenario, we measure overall ordering through the quantity called *efficiency*:

$$E\left(t\right)=\frac{1}{Nv_{0}}\left|\sum_{i=1}^{N}{\vec{v}}_{i}\left(t\right)\right|.$$ (2)

Note that in theory, Eq 1 does allow for *E*(*t*) to exceed 1, if many agents have $|{\vec{v}}_{i}|>1$. However, in practice, even with 2 agents this is a very rare case. For *N* = 200 agents Fig 4 shows the efficiency in 30 single simulation runs and also the average over all 30 runs. Observe in this figure that the formation of small stable flocks (slightly elevated values of the efficiency compared to the initial level) is quickly followed by the stable ordering of all agents (*E* moves quickly up close to 1). In other words, for an individual system the transition from disorder to order is *fast*, and the slowness of the ordering (growth) seen on the averaged curve is caused merely by the wide temporal distribution of the fast transitions of the individual systems.

**Fig 5:**
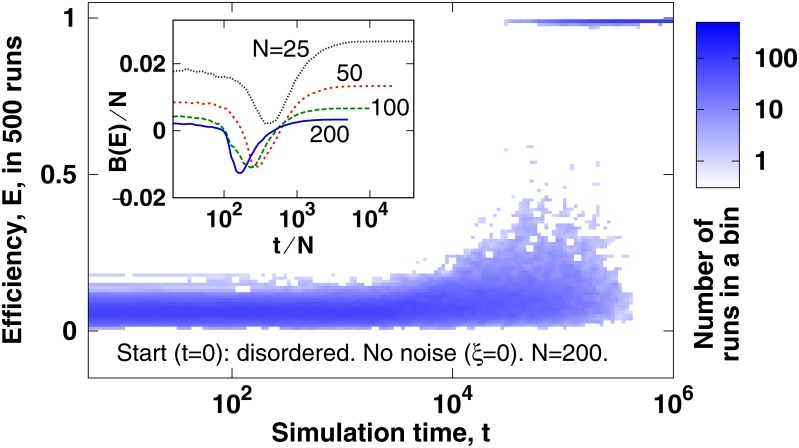
Main panel. Ordering of *N* = 200 particles started from the disordered state 500 times randomly. Pixel color depths show the number of independent simulation runs that have an efficiency within a given interval at the given simulation time point. The ratio of adjacent time values is 10^1/30^, and the width of *E* intervals is 0.01. **Inset**. The normalized Binder cumulant, *B*(*E*)/*N*, of the distribution of the 500 values of *E*(*t*). shows that the transition remains bimodal (i.e., discontinuous) with increasing system size. With changing *N* we change *L* also to keep the density of particles—*N* × *L*^−1/3^—constant. See text for the definition of *B* and further details.

For the same transition Fig 5 shows an alternative view with a much larger data set. Here we evaluate 500 simulation runs (instead of the previous 30) to obtain the distribution of these 500 efficiency values at each of the selected time points. Similar to Fig 4, we observe on the main panel of Fig 5 that between the simulation times *t* = 10^4^ and 10^6^ each system moves relatively quickly from the disordered state (0 ≤ *E* ≪ 1) to the ordered state (*E* ≈ 1). Regarding transitions, a frequently investigated question is how the type of the transition changes with increasing system size [33, 34]. In the inset of Fig 5 we quantify the distribution of efficiency values at the selected simulation time points by computing the Binder cumulant, *B*(*E*), of the numerically measured *E* values:

$$B\left(E\right)=\frac{1-\langle E^{4}\rangle }{3{\langle E^{2}\rangle }^{2}}.$$ (3)

We find that growing system size (and constant density) the transition from the ordered to the disordered state remains rapid. For practical applications this means that—in the absence of disturbances—once the transition to the ordered state starts, it is very hard to stop, and the resulting ordered state is very stable. To go into more detail, in the following we investigate the stability of the arising ordered state to two of the most common disturbances: noise (errors) and delay (also called time lag).

### Stability of the ordered state to noise

**Fig 6:**
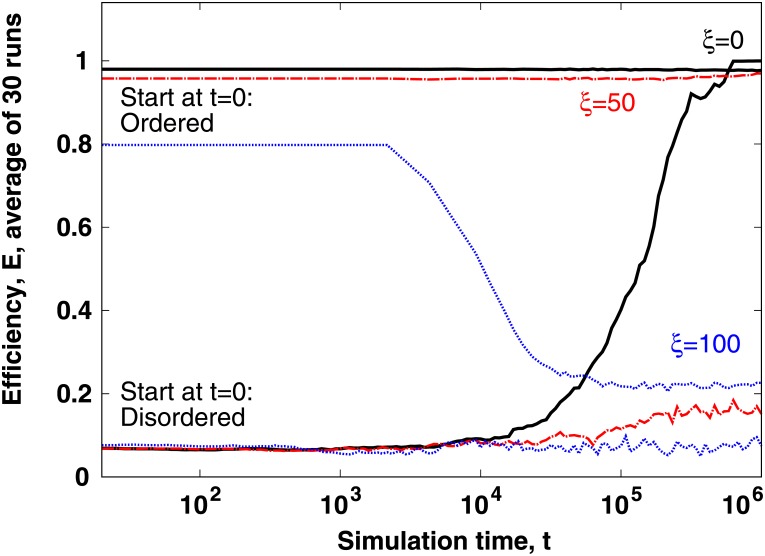
According to the current model, the lowest amount of noise that can keep the system disordered is not sufficient for destroying order. This property—called hysteresis—makes order very stable even at intermediate noise levels. On the other hand, it also implies that a transition started at intermediate noise levels is very fast. Parameters are as in Sec. Here the ordered state was measured after running (equilibrating) the simulation for 10^5^s. See text for details.

Errors and small changes (also called fluctuations or noise) are ubiquitous in natural and social phenomena. In most cases, a low amount of noise still allows ordering, however, when noise amplitudes grow very large, order is destroyed and the system becomes disordered. A known exception to this rule is the phenomenon of “freezing by heating”, when—compared to the case without noise—an intermediate amount of noise can lead to a new ordered structure [35]. In Fig 6 we test whether it is easy to destroy the ordered state (high *E*) of the agents of Eq 1 by increasing the initially low amount of noise to a high value. With the parameters selected in Sec we find that the noise level *ξ* = 50 can already keep an initially disordered system from reaching order, however, it is not sufficient to destroy order in an initially ordered system. We conclude that the system shows hysteresis, which is similar to the 2-dimensional version of the model [32]. Finally, note that this behavior stabilizes the ordered state, and at the same time makes transitions fast.

In this final paragraph of Sec, we add a note regarding how we start the system from the ordered state. For the curves labeled “Start: ordered” in Fig 6 we start each of the 30 simulations (with 30 different random seeds) at *t*_start_ = −100,000*s* by setting the velocity vectors of the agents fully aligned and their coordinates randomly with a minimal distance. Here minimal initial distance means—similar to the case of the disordered initialization in Sec—a lower bound of 0.6*LN*^−1/3^ for the agent-agent distance. First, starting at *t*_start_, we run the simulation until *t* = 0*s* to reach spatial ordering. After the equilibration interval that starts at *t*_start_ and ends at *t*_0_, we run the numerical integration of the equations of motion until *t* = 10^5^ or *t* = 10^6^. Note that in Sec we do not apply the equilibration technique described here.

### Stability of the ordered state to delay (time lag)

In most natural, social and technological phenomena the delays of interactions play a crucial role in shaping the emerging collective behavioural patterns. Stable formations of collective motion can usually emerge only if the distance traveled by an agent over the delay time interval with its characteristic relative speed (relative to nearby agents and obstacles) remains safely below the agent’s distance to those other agents and obstacles. For the current model we set a single delay time interval, *t*_*d*_, that affects in an identical way how an agent responds to any internal or external effect. For the numerical integration, we implement delayed interactions by setting the time lag, *t*_*d*_, of all effects (forces) to a multiple of the numerical integration time step, Δ*t*. Next, we replace all forces on the right hand side of Eq 1 by the values of the same forces measured *t*_*d*_ time before the current time. Note that these forces include self-propelling, therefore, a particle’s response to changes in its own speed are delayed as well.

**Fig 7:**
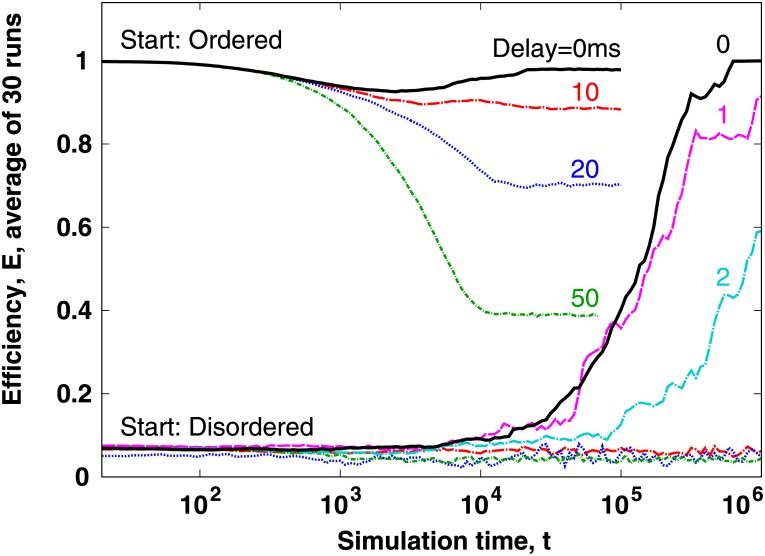
Time delays (time lags) introduced to the interactions of the model affect the ordered and the disordered states differently. During the emergence of order, short time lags (even 1 or 2 ms) can significantly change the rate of ordering. On the other hand, for highly diverse values (up to 50ms) of the time lag order is destroyed on the same time scale. The time delay values of the interactions are shown in milliseconds over the respective measured average curves. Note that at the start of the simulation the efficiency drops slightly and then grows again to reach *E* ≈ 1. To explain this effect, recall that this simulation is started with directionally ordered agents that are spatially not yet ordered. The slight drop of the efficiency shows as the particles attain their spatial order (close to crystalline ordering of coordinates) while temporarily losing some of their directional alignment.

We find that—similar to noise—interaction delays act differently on the ordered and disordered states of the system (see Fig 7). First, the two states respond to time delays at different time scales. Whereas the ordered state responds to a wide range of interaction delays on the same short time scale, the already slow disorder → order transition is further significantly delayed even by small amounts of the interaction delay. Second, even large amounts (up to *t*_*d*_ = 50ms) of the interaction delay cannot entirely destroy the ordered state. Third, based on our current results with *t*_*d*_ = 0, 10, 20 and 50, we conclude that ordering is lost as a linear function of the amount of interaction time lag, *t*_*d*_.

## Discussion and outlook

In this paper, we investigated a minimal continuous model of 3-dimensional collective motion. The model contains continuous adjustment of particle speed to a preferred value, pairwise radial repulsion for collision avoidance, and an effective weak attraction (periodic boundaries). We found that the combination of these three model components is sufficient for stable spatial ordering, and beyond these three no further model components are necessary. After investigating the model on the microscopic level, we found that for the majority of symmetric two-agent encounters the total momentum of the two agents increases. Regarding the macroscopic level, we found that the transition from disorder to order is fast for both small and large system sizes, which is in good agreement with previous results [27]. In the minimal continuous model that we investigated we found also that if the noise intensity is above a threshold value, then the system cannot reach the ordered state, similarly to results reported in [36].
